# The relationship between mothers’ attachment style, mindful parenting, and perception of the child

**DOI:** 10.1192/j.eurpsy.2022.893

**Published:** 2022-09-01

**Authors:** B. Szabó, M. Miklósi

**Affiliations:** 1 Eötvös Loránd University, Department Of Developmental And Clinical Child Psychology, Budapest, Hungary; 2 ötvös Loránd University, Department Of Developmental And Clinical Child Psychology, Budapest, Hungary

**Keywords:** mindful parenting, maternal attachment, child perception, mindfulness

## Abstract

**Introduction:**

Maternal attachment style plays a major role in the intergenerational transmission of psychopathology. Previous studies indicated that a secure attachment style is associated with higher levels of mindfulness and a higher quality of the parent-child relationship.

**Objectives:**

The aim of this study was to explore the relationship between the mothers’ attachment style, mindful parenting, and perception of the child.

**Methods:**

Data was collected from 144 non-clinical mothers, who have a child below the age of 3 years. Mothers completed self-report questionnaires including the following scales: a demographic questionnaire, Attachment Style Questionnaire (ASQ), Interpersonal Mindfulness in Parenting Scale (IMP), and the Mothers’ Object Relations Scale (MORS-SF). Mediation analyses with Mothers’ Object Relations Scale warmth and invasion subscales as dependent variables, mother’s attachment style as an independent variable and, mindful parenting as a mediator were conducted.

**Results:**

In mediation analysis, the direct effects of the mothers’ attachment style on the perception of the child were not significant. However, indirect effects through mindful parenting were significant; higher levels of mindful parenting were associated with higher levels of MORS-SF warmth and lower levels of MORS-SF invasion.

**Conclusions:**

These findings suggest that attachment styles are related to the perception of the child through mindful parenting. Mindfulness-based parenting training might be useful in case of attachment-related problems to improve the parent-child relationship.

**Disclosure:**

No significant relationships.

